# Silencing of the chemokine CXC receptor 4 (CXCR4) hampers cancer progression and increases cisplatin (DDP)-sensitivity in clear cell renal cell carcinoma (ccRCC)

**DOI:** 10.1080/21655979.2021.1943112

**Published:** 2021-06-28

**Authors:** Wenguang Wang, Zhilu Gan, Qiang Liu, Shenshen Yan, Rexiti Mulati, Yujie Wang

**Affiliations:** a Department of Urology, The First Affiliated Hospital of Xinjiang Medical University, Urumqi, China; b Department of Urology, The Third People’s Hospital of Xinjiang Uygur Autonomous Region, Urumqi, China

**Keywords:** Clear cell renal cell carcinoma, chemokine receptor 4, cisplatin-sensitivity, cancer biology

## Abstract

Aberrant expression of the chemokine CXC receptor 4 (CXCR4) is closely associated with cancer progression and drug-resistance in multiple cancers, and we first investigated the role of CXCR4 in regulating cancer pathogenesis and cisplatin (DDP)-resistance in clear cell renal cell carcinoma (ccRCC) in the present study. Here, we identified that CXCR4 acted as an oncogene to promote cancer progression and genetically silencing of CXCR4 increased cisplatin (DDP)-sensitivity in ccRCC *in vitro* and *in vivo*. Functionally, analysis from the clinical and cellular data indicated that CXCR4 was significantly upregulated in ccRCC tissues and cells, compared to their normal counterparts. Next, the loss-of-function experiments validated that knock-down of CXCR4 suppressed cell proliferation, invasion, migration and epithelial-mesenchymal transition (EMT) in ccRCC cells, while CXCR4 overexpression had opposite effects on the above cellular functions. Consistently, the xenograft tumor-bearing mice models were established, and the results supported that knock-down of CXCR4 inhibited tumor growth and the expression levels of Ki67 protein *in vivo*. In addition, the ccRCC cells were exposed to DDP treatment, and we surprisingly found that upregulation of CXCR4 increased DDP-resistance in ccRCC cells, and conversely, CXCR4 ablation sensitized ccRCC cells to DDP stimulation. Taken together, we concluded that CXCR4 ablation hindered cancer progression and enhanced DDP-sensitivity in ccRCC, and the present study identified a novel therapeutic biomarker for ccRCC.

## GRAPHICAL ABSTRACT

**Figure:**
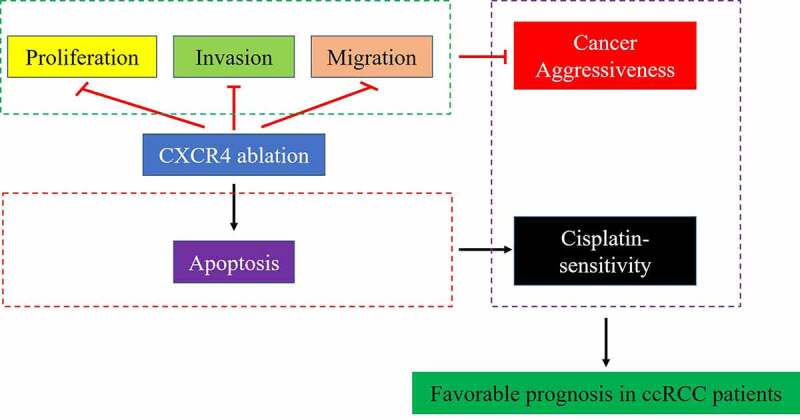

## Introduction

Clear cell renal cell carcinoma (ccRCC) is a common malignancy in kidney, which accounted for about 85% of all the renal-associated cancers and 2–3% of total adult malignancies [1], and the morbidity and mortality of ccRCC rank third among all the tumors that occur in the urinary system [2,3]. Although great advances have been reached in developing novel cancer therapies, surgery remains the unique effective treatment strategy for ccRCC [4,5]. The main reason is that ccRCC cells always generate resistance to other types of cancer treatments, such as radiotherapy, chemotherapy, and immunotherapy [4,5]. According to the data in 2019 [6,7], as the results of its metastatic properties, about 30% of ccRCC patients develop metastatic RCC (mRCC) after surgery [6,7], leading to the worse outcome and seriously degrades the life quality of mRCC patients, and researchers agree that uncovering the underlying mechanisms of ccRCC pathogenesis may help to improve the efficacy of current therapies [8–10]. Based on this, various cancer associated genes, including tumor suppressors and oncogenes, have been identified in ccRCC [11,12].

Recent data report that chemokines and their receptors involve in regulating cancer progression by aggravating the malignant phenotypes of cancer cells, such as cell proliferation, angiogenesis, invasion, migration and tumor immune cells penetration [13]. Among all the chemokine receptors, emerging evidences suggest that the chemokine CXC receptor 4 (CXCR4) is closely related with the development of multiple cancers, such as breast cancer [13,14], colorectal cancer [15], gastric cancer [16] and adrenocortical cancer [17]. Specifically, CXCR4 participates in the regulation of cancer progression by regulating both tumor immune-microenvironment [18], cancer-associated fibroblasts [19] and cancer cell functions [13,14,17,20]. Of note, overexpression of CXCR4 is also associated with poor survival and worse prognosis in ccRCC [21–24], and the antagonist of CXCR4 reversed the tumor-promoting microenvironment in renal cancer [25], indicating that CXCR4 may be a theranostic target for ccRCC. In addition, the underlying mechanisms by which CXCR4 involved in regulating cancer progression are complicated, and competing endogenous RNA (ceRNA) mechanisms may be the reason [26].

Generation of drug resistance has become a serious obstacle to degrade the therapeutic efficacy of current chemical drugs for ccRCC treatment in clinic, such as cisplatin (DDP) [27–29]. According to the existed information, DDP-other types of treatments (other chemical drugs, radiotherapies, and immunotherapies) combined treatment strategies have been used for ccRCC treatment [27,28,30–32]. Unfortunately, researchers notice that aberrant expressions of some cancer-associated genes contribute to DDP-resistance in ccRCC treatment, and targeting the corresponding genes, including CIP2A [30], SMYD2 [28], and PARK7 [27], improve DDP-sensitivity in ccRCC cells. Interestingly, chemokine receptors regulate DDP-sensitivity [33–35], and CXCR4 is identified as a crucial regulator to enhance DDP-resistance in human tongue squamous cell carcinoma [36] and sunitinib-resistance in metastatic renal cancer [37]. Based on the above information, we first proposed that CXCR4 might regulate DDP-sensitivity in ccRCC.

In summary, we aimed to investigate the role of CXCR4 in regulating cancer progression and drug resistance in ccRCC *in vitro* and *in vivo*, which not only broadened our knowledge in this field but provided evidences to support that CXCR4 could be used as a feasible biomarker for ccRCC diagnosis and prognosis.

## Materials and methods

### Collection of the clinical specimens

The clear cell RCC (ccRCC) patients (N = 13) were recruited in the First Affiliated Hospital of Xinjiang Medical University from 2015 to 2018, and the ccRCC tissues and the corresponding adjacent non-cancerous normal tissues were obtained by surgical resection and immediately restored at −80°C conditions. All the patients did not accept any other adjuvant therapies, including chemotherapy and radiotherapy, before surgery. We had obtained the signed informed consent forms from all the patients, and all the clinical experiments were in keeping with the Helsinki Declaration and were approved by the Ethics Committee of the First Affiliated Hospital of Xinjiang Medical University.

### Cell culture, treatment and vectors transfection

The human ccRCC cell lines (SW839 and OSRC-2) and the human normal kidney epithelial cell-line HK2 were obtained from American Type Culture Collection (ATCC, USA), and the above cells were maintained in the Dulbecco’s modified Eagle’s medium (DMEM, Gibco, USA) in the incubator with 5% CO_2_ humidified atmosphere and 37°C. The CXCR4 overexpression and downregulation fluorescent vectors were designed and synthesized by Sangon Biotech (Shanghai, China), and were delivered into the ccRCC cells by using the Lipofectamine 2000 transfection reagent (Invitrogen, USA) according to the protocols provided by the manufacturer, the fluorescent microscope was used to assure the successful transfection of the vectors. Then, the ccRCC cells were subjected to 30 μg/ml for 0 h, 24 h and 48 h, and were prepared for further analysis. The sequences for CXCR4 overexpression and downregulation were designed according to the previous work [26,38].

### Real-Time qPCR

The total RNA was extracted from the ccRCC tissues and cells by using the Trizol reagent purchased from Ambion (USA) following the protocols provided by the manufacturer. A NanoDrop spectrophotometer (ThermoFisher Scientific, USA) was then used to quantify the total RNA, which were reversely transcribed into cDNA by using the commercial Transcriptor First Strand cDNA synthesis kit (Roche Diagnostics, Switzerland), and agarose gel electrophoresis was conducted to ensure that we had successfully obtain the total cDNA. The mRNA levels for CXCR4 were examined by using the SYBR green I (Roche Diagnostics, Switzerland), which were normalized by the internal reference GAPDH. The primer sequences for CXCR4 (Forward: 5ʹ-GGA GGG GATCAG TAT ATA CA-3ʹ, Reverse: 5ʹ-GAA GATGATGGAGTAGATGG) and GAPDH (Forward: 5ʹ-ATG TTC GTC ATG GGT GTG AA-3ʹ).

### Western Blot

The clinical tissues and cells were prepared, and the RIPA lysis buffer (Beyotime Biotechnology, Shanghai, China) was employed for total proteins extraction. Next, we conducted SDS-PAGE to separate the protein bands with differential molecular weight, and the proteins were transferred onto the PVDF membranes (Millipore, USA). Then, the membranes were blocked by nonfat milk for 1 h at room temperature, and were incubated with the primary antibodies anti-CXCR4 (#ab181020, 1:2000, 39 kDa, Abcam, UK), anti-GAPDH (#ab8245, 1:3000, 40.2 kDa, Abcam, UK), anti-N-cadherin (#ab207608, 1:2500, 100 kDa, Abcam, UK), and anti-Vimentin (#ab137321, 1:1500, 54 kDa, Abcam, UK) at 4°C overnight. Next day, the membranes were probed with the an-mouse and anti-rabbit secondary antibodies (Cell Signaling Technology, USA) for 1 h with the dilution of 1:5000, and the protein bands were visualized by ECL chemiluminescent detection system (ThermoFisher Scientific, USA) and were quantified by Image J software. The proteins were normalized by GAPDH.

### Examination of cell proliferation and viability

The commercial cell counting kit-8 (CCK-8) kit (YEASEN, Shanghai, China) and trypan blue staining solution (LEAGENE, Beijing, China) were purchased to examine cell proliferation abilities and viability, respectively. For the CCK-8 assay, the ccRCC cells were maintained in the 96-well plates at the density of 1000 cells per well, and at 0 h, 24 h, 48 h and 72 h post-culture, the cells were incubated with the CCK-8 reaction solution for 2 h at 37°C, and the plates were vortexed and the optical density (OD) values were examined by using the microplate reader (ThermoFisher Scientific, USA) at the wavelength of 450 nm. For the trypan blue staining assay, the cells were stained with the trypan blue staining dye for 30 min at 37°C, and the dead blue cell numbers were counted. Cell viability was calculated by using the following formula: Cell viability (%) = (total cells – dead blue cells)/total cells × 100%.

### Examination of cell migration

We performed transwell assay and wound scratch assay to examine cell migration abilities in the ccRCC cells. For the transwell assay, the ccRCC cells were maintained in the upper chambers of the transwell plates (Corning, USA) with 200 μl FBS-free DMEM medium at the density of 2 × 10^5^ cells per well, and the lower chambers were added with 500 μl DMEM containing 10% FBS, which was used as chemoattractant. At 24 h post-culture at the standard culture conditions, the cells in the upper surface of the Matrigel were removed, and the cells in lower surface were stained with 0.1% crystal violet solution (Sigma-Aldrich, USA) for 20 min at room temperature, and the light microscope (ThermoFisher, USA) was used to observe and count cell numbers in the lower surface. For the wound scratch assay, the ccRCC cells were maintained in the 6-well plates at the concentration of 4 × 10^5^ cells per well, until the cell confluency reached about 95%, the 100 μl tip were used to generate the scratches, and the scratch distances were monitored at 0 h and 24 h, respectively.

### Flow cytometer analysis for cell apoptosis

The commercial apoptosis detection kit (YEASEN, Shanghai, China) was purchased to examine cell apoptosis in ccRCC cells according to the manufacturer’s protocol. Briefly, the ccRCC cells were subjected to vectors transfection (48 h post-delivery) and cisplatin administration (30 μg/ml for 24 h), and were double-stained with Annexin V-FITC and PI for 40 min at room temperature without light exposure. Then, a FACSVerse flow cytometer (Becton-Dickinson, USA) was employed to examine cell apoptosis status in ccRCC cells.

### In vivo *animal experiments*

The male nude mice (6–8 weeks) were purchased from the Research Animal Center of Xinjiang Medical University, and were fed in the specific-pathogen-free (SPF) conditions. All the mice were freely accessible to food and water, the ccRCC cells with CXCR4 overexpression and deficiency were subcutaneously injected into the dorsal flank of the mice for tumor formation, and at day 25 post-injection, the mice were injected with 50 mg/kg of pentobarbital sodium for anesthetization. Then, the mice were sacrificed and the tumors were obtained, weighed and prepared for further analysis.

### Immunohistochemistry (IHC) assay for Ki67 protein staining

The mice tumor tissues were prepared and fixed by using the 10% (v/v) formaldehyde, which were diluted by using the PBS buffer. Then, the tumor tissues were embedded into the paraffin, and were sliced into sections with 5 μm thickness. Then, the IHC assay was conducted to examine the expression levels and localization of Ki67 protein in the tissues, and the primary antibody against Ki67 was purchased from Cell Signaling Technology (USA). Finally, a light microscope was employed to evaluate Ki67 protein status in mice tissues.

### Data analysis

The SPSS 11.5 software was used to analyze the data in this study. Specifically, comparisons between two groups were conducted by using the Student’s t-test, and the one-way ANOVA analysis was used to compare the data from multiple groups (>2). The GraphPad Prism 5.0 software was employed for data visualization. *P< 0.05 indicated statistical significance.

## Results

### Aberrant expressions of CXCR4 in ccRCC tissues and cells

**Figure 1.**
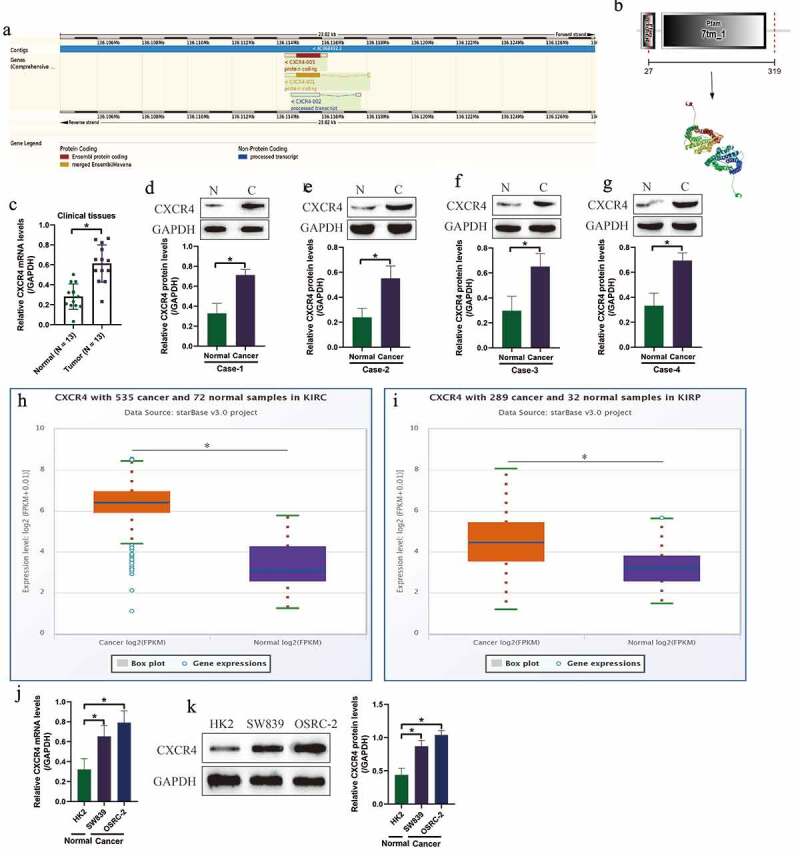
Characteristics and expression status of CXCR4 in ccRCC clinical tissues and cells. (a) The gene sequence/location information and (b) homology model for CXCR4. (c) The mRNA levels and (d-g) protein levels of CXCR4 were examined by using the Real-Time qPCR and Western Blot analysis. The expression status of CXCR4 in (h) KIRC and (i) KIRP were analyzed by using the Pan-cancer analysis software (http://starbase.sysu.edu.cn/panCancer.php). (j) Real-Time qPCR and (k) Western Blot were used to transcriptionally and translationally examined the levels of CXCR4 in ccRCC cells. Individual experiment was repeated 3 times, and * *P*< 0.05

Although previous data suggested that aberrant expressions or mutations of CXCR4 are relevant to cancer development [13–17], but the detailed regulating mechanisms have not been elucidated. According to the public STRING database (https://string-db.org/), CXCR4 gene located in the chr2: 136, 114, 349–136, 118, 165, which contained 3 transcripts (Figure 1(a)) and the homology model for CXCR4 is shown in Figure 1(b). In addition, we initially examined the expression status of CXCR4 in ccRCC tissues (Figure 1(c-i)) and cell lines (Figure 1(j-k)). Specifically, the 13 paired ccRCC tissues and normal non-cancerous tissues were collected, and the Real-Time qPCR results in Figure 1(c) showed that CXCR4 mRNA was upregulated in cancer tissues, compared to their normal counterparts. Consistent with this, further Western Blot analysis validated that CXCR4 protein levels were also increased in the cancer tissues (Figure 1(d-g)). Next, by conducting the online Pan-cancer analysis (http://starbase.sysu.edu.cn/panCancer.php), we found that CXCR4 was significantly upregulated in the cancer tissues collected from patients with kidney renal clear cell carcinoma (KIRC) (Figure 1(h)) and kidney renal papillary cell carcinoma (KIRP) (Figure 1(i)). Then, the human ccRCC cell lines (SW839 and OSRC-2) and the human normal kidney epithelial cell-line HK2 were obtained, and analysis of the data in Figure 1(j,k) supported that CXCR4 tended to be high-expressed in the ccRCC cells, compared to the normal HK2 cells.

### *CXCR4 promoted cell growth* in vitro *and* in vivo

**Figure 2.**
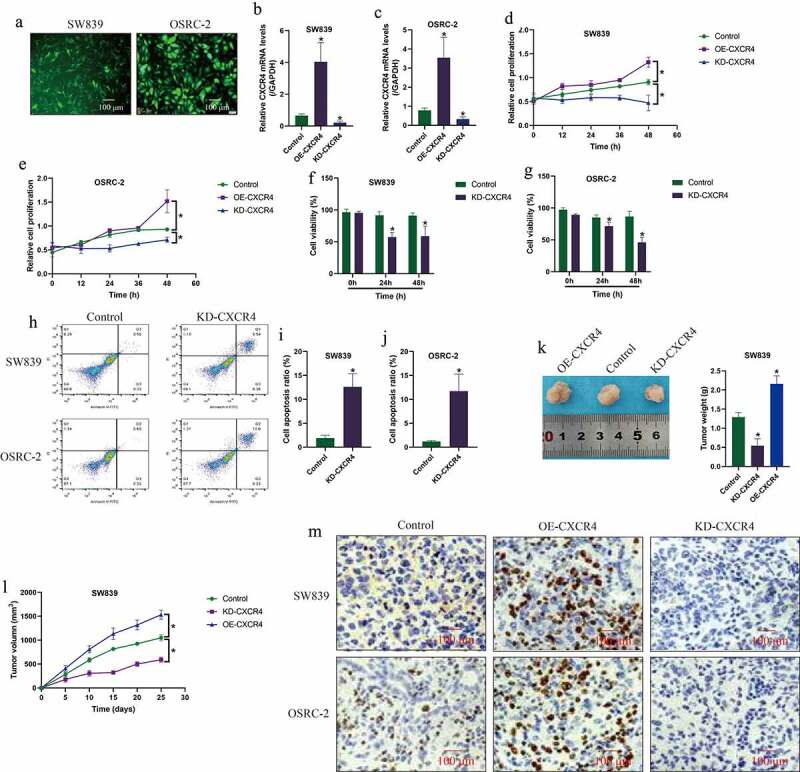
CXCR4 positively regulated cell growth, viability and tumorigenesis in ccRCC cells *in vitro* and *in vivo*. (a) Fluorescence staining assay observed that the vectors were successfully delivered into the ccRCC cells, which were validated by the following Real-Time qPCR analysis in the (b) SW839 and (c) OSRC-2 cells. (d-e) Cell proliferation and (f-g) cell viability was examined by CCK-8 assay and trypan blue staining assay. (h-j) Cell apoptosis ratio was determined by FCM assay. (k) Tumor weight and (l) volume were respectively monitored during 25 days tumor growth *in vivo*. (m) CXCR4 promoted Ki67 protein expressions in mice tumor tissues, examined by immunohistochemistry (IHC). Individual experiment was repeated 3 times, and * *P*< 0.05

Next, the CXCR4 overexpression and downregulation vectors were transfected into the ccRCC cell lines (SW839 and OSRC-2) for further gain- and loss-of-function experiments to investigate the regulating effects of CXCR4 on ccRCC progression. The fluorescence images in Figure 2(a), Real-Time qPCR results in Figure 2(b,c) and Western Blot results in Figure S1A-B validated that the above vectors had been successfully delivered into the cells. Next, cell proliferation abilities and viability were monitored by using the CCK-8 assay and trypan blue staining assay for 0 h, 12 h, 24 h, 36 h and 48 h, and the results in Figure 2(d,e) showed that overexpression of CXCR4 promoted cell proliferation in the ccRCC cells, while CXCR4 ablation had opposite effects to hinder RCC cell growth (Figure 2(d,e)) and inhibited cell viability (figure 2(f,g)). Next, the FCM assay was performed to determine cell apoptosis, and we expectedly found that silencing of CXCR4 increased Annexin V-FITC/PI-positive apoptotic cell ratio in the ccRCC cells (Figure 2(h-j)). Finally, the SW839 cells with CXCR4 deficiency and overexpression were used to establish xenograft tumor-bearing mice models, and the tumor weight (Figure 2(k)) and volume (Figure 2(l)) were, respectively, monitored. The results in Figure 2(k-l) showed that CXCR4 positively regulated tumor growth in mice models *in vivo*. Consistently, the immunohistochemistry (IHC) was performed, and the results validated that CXCR4 positively regulated Ki67 protein levels in mice tumor tissues (Figure 2(m)).

### The regulating effects of CXCR4 on cell migration and epithelial-mesenchymal transition (EMT)

**Figure 3.**
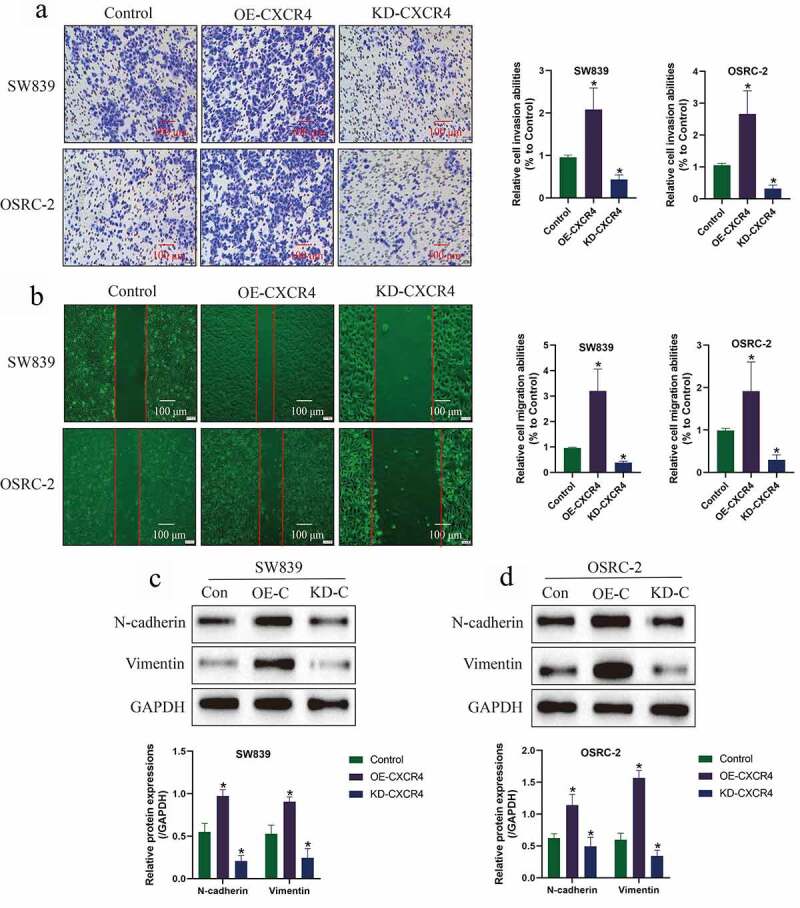
CXCR4 promoted cell invasion, migration and EMT in ccRCC cells. (a) Cell invasion and (b) migration were examined by Transwell assay and wound scratch assay, respectively. (c-d) Western Blot was used to examine the expression levels of EMT associated markers (N-cadherin and Vimentin) in ccRCC cells. Individual experiment was repeated 3 times, and * *P*< 0.05

Based on the published information that CXCR4 participates in the regulation of cancer metastasis, we explored whether CXCR4 also had similar regulating effects on the ccRCC cells. To investigate this issue, the ccRCC cell lines (SW839 and OSRC-2) with CXCR4 overexpression and ablation were employed, and the transwell assay (Figure 3(a)) and wound healing assay (Figure 3(b)) were performed to examine cell invasion and migration, respectively. As shown in Figure 3(a,b), we validated that knock-down of CXCR4 significantly inhibited cell invasion (Figure 3(a)) and migration (Figure 3(b)) in ccRCC cells, which were significantly promoted by upregulating CXCR4. Also, the Western Blot analysis was conducted, and the results supported that CXCR4 positively regulated N-cadherin and Vimentin (Figure 3(c,d)), while negatively regulated E-cadherin (Figure S2A-B) to promote epithelial-mesenchymal transition (EMT) in ccRCC cells.

### *CXCR4 regulated DDP-sensitivity in ccRCC cells* in vitro

**Figure 4.**
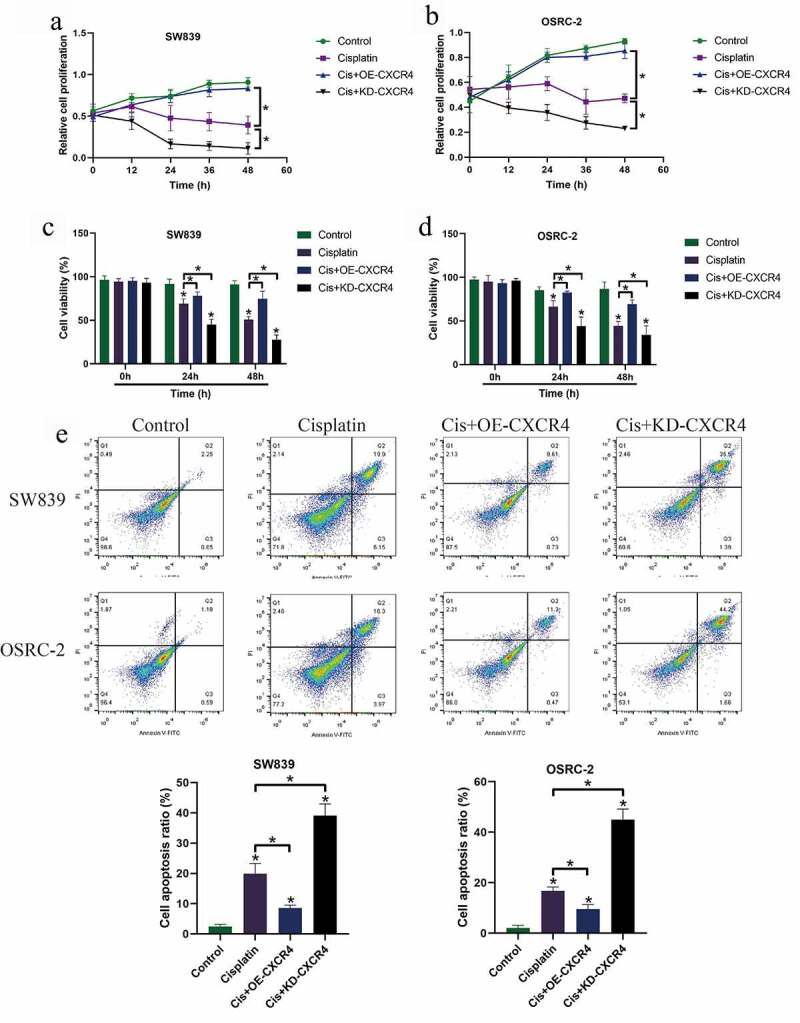
Silencing of CXCR4 increased susceptibility of ccRCC cells to DDP treatment. (a-b) CCK-8 assay was used to examine cell proliferation, and (c-d) cell viability was determined by trypan blue staining assay. (e) FCM analysis was conducted to examine the apoptosis ratio in ccRCC cells. Individual experiment was repeated 3 times, and * *P*< 0.05

**Figure 5.**
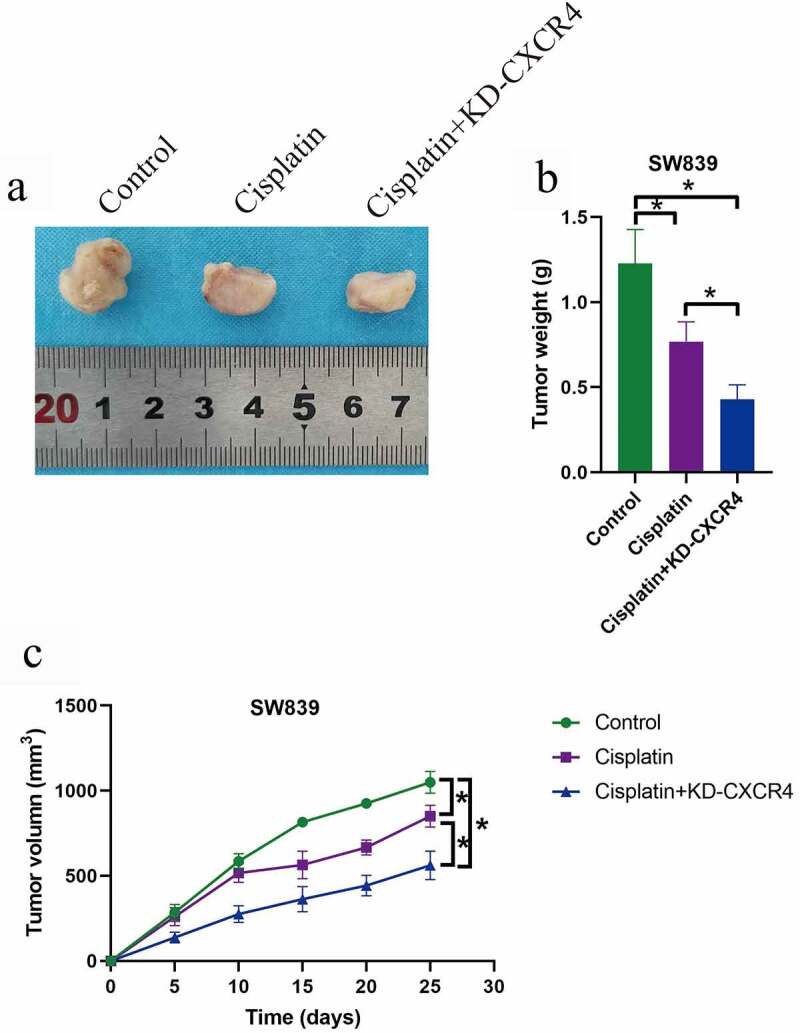
CXCR4 ablation enhanced the inhibiting effects of cisplatin on tumor growth *in vivo*. (a, b) Tumor weight and (c) volume were respectively monitored during 25-days tumor growth. Individual experiment was repeated 3 times, and * *P*< 0.05

Cisplatin (DDP) has been used for ccRCC treatment [27,28,30–32], but DDP-resistance generated by the RCC cells seriously limits the therapeutic efficacy of this drug in clinic. Recent data suggested that targeting the cancer associated genes was proved as novel strategies to improve drug sensitivity [8–12], but the role of CXCR4 in regulating cisplatin-sensitivity has not been investigated. Here, the ccRCC cells (SW839 and OSRC-2) were exposed to DDP (30 μg/ml) for 0 h, 12 h, 24 h, 36 h and 48 h, and were divided into four groups, including Control, DDP group, DDP + OE-CXCR4 group, and DDP + KD-CXCR4 group. As shown in Figure 4(a-d), the CCK-8 assay and trypan blue staining assay results showed that DDP suppressed cell proliferation and viability in a time-dependent manner, which were reversed by upregulating CXCR4 and aggravated by downregulating CXCR4. In addition, the FCM results in Figure 4(e) supported that upregulation of CXCR4 decreased, while CXCR4 ablation increased cell apoptosis ratio in ccRCC cells treated with DDP. Furthermore, by performing the in vivo experiments, we evidenced that knock-down of CXCR4 enhanced the suppressing effects of cisplatin on tumor weight (Figure 5(a,b)) and volume (Figure 5(c)) in tumor-bearing mice models established by using the SW839 cells.

## Discussion

As the results of its complicated pathogenesis, the current therapies for clear cell renal cell carcinoma (RCC) treatment have been seriously limited [4,5], and researchers notice that targeting the cancer associated genes, including tumor suppressors and oncogenes, is effective to hamper cancer progression and improve the therapeutic efficacy of radio- or chemo-therapy in ccRCC [8–12]. Among all the genes, the chemokine CXC receptor 4 (CXCR4) is identified as an oncogene to facilitate the development of multiple cancers [13–17], and recent data indicate that CXCR4 was closely related to patients’ prognosis in ccRCC [21–24], but its role in regulating RCC development and the potential underlying mechanisms have not been investigated in the available publications. Here, we noticed that CXCR4 tended to be enriched in ccRCC tissues and cell lines, compared to their normal counterparts, which were supported by the Pan-cancer analysis results in kidney renal clear cell carcinoma (KIRC) and kidney renal papillary cell carcinoma (KIRP), indicating that aberrantly expressed CXCR4 was closely associated with ccRCC progression, which were in accordance with the previous literatures [21–24].

According to the previous publications [21–24], CXCR4 positively regulates the malignant phenotypes to facilitate cancer progression, which were validated by our results in this study. To achieve this, the CXCR4 was overexpressed and silenced in ccRCC cells, respectively. Further gain- and loss-of-function experiments evidenced that overexpression of CXCR4 promoted cell proliferation, viability, migration, epithelial-mesenchymal transition (EMT) and tumor formation *in vitro* and *in vivo*, while knock-down of CXCR4 had opposite effects on the above cellular functions and induced ccRCC cell apoptosis, implying that CXCR4 promoted RCC progression, which were supported by the previous literatures in other types of cancer [39–42]. Specifically, data from different laboratories evidence that CXCR4 exerts its tumor-promoting effects to accelerate cancer cell proliferation, migration and invasion in ovarian cancer [42], glioblastoma [41], colorectal cancer [40], and prostate cancer [39]. In addition, since CXCR4 transduces a signal by increasing intracellular calcium ion levels and enhanced the activation of MAPK1/MAPK3 pathway [43,44], and activating the MAPK1/MAPK3 signaling pathway enhanced cancer progression in ovarian cancer [45], we conjectured that CXCR4 might activated the MAPK1/MAPK3 pathway to facilitate ccRCC development. However, future work is needed to validate this hypothesis.

Cisplatin (DDP) has been used as therapeutic chemical drug for ccRCC treatment, and DDP-other types of therapies combined treatment strategies have been widely used for ccRCC in clinic [27,28,30–32]. Unfortunately, researchers noticed that long-term DDP exposure altere the expression patterns of some cancer-associated genes, resulting in the survival of DDP-resistant subgroups of ccRCC cells, and expansion of those cells makes ccRCC cells resistant to further DDP treatment [27,28,30]. Given that CXCR4 acts as an oncogene in ccRCC [39–42], and previous data suggested that CXCR4 also regulated drug resistance in cancer treatment [36,37], we validated that targeting CXCR4 was effective to improve DDP-sensitivity in ccRCC cells in the present study. Specifically, the CXCR4 overexpression and downregulation vectors were delivered into the RCC cells, which were subsequently treated with high-dose DDP. The results showed that silencing of CXCR4 enhanced the promoting effects of DDP on cell apoptosis and death in ccRCC cells, while CXCR4 overexpression had opposite effects and rescued cell viability in ccRCC cells, indicating that CXCR4 increased DDP-resistance, which were partially supported by the previous studies [36,37].

## Conclusions

Based on the presented data, we concluded that, on the one hand, CCR4 acted as an oncogene and promoted cancer cell proliferation, migration, epithelial-mesenchymal transition (EMT) and tumorigenesis, to facilitate cancer progression in ccRCC. On the other, genetically silencing of CCR4 improved cisplatin-sensitivity in ccRCC cells. In general, this study identified the role of CCR4 in regulating ccRCC progression and cisplatin-resistance, and CCR4 could be used as a theranostic biomarker for RCC.

## Supplementary Material

Supplemental Material

## Data Availability

All the associated data had been included in the manuscript.
