# Supplementary material for: Silencing of the chemokine CXC receptor 4 (CXCR4) hampers cancer progression and increases cisplatin (DDP)-sensitivity in clear cell renal cell carcinoma (ccRCC)
Source: Bioengineered. 2021 Jun 28;12(1):2957–69. doi: 10.1080/21655979.2021.1943112 (PMC8806489; doi:10.1080/21655979.2021.1943112)
Supplement: Supplemental Material [file KBIE_A_1943112_SM1365.zip › Supplementary Figure legends.docx]

**Supplementary Figure legends**

**Figure S1.** Western Blot was performed to determine the transfection efficiency of CXCR4 in SW839 and OSRC-2 cells. Individual experiment was repeated 3 times, and * *P* < 0.05.

**Figure S2.** The expression status of E-cadherin in the ccRCC cells were measured by performing Western Blot analysis. Individual experiment was repeated 3 times, and * *P* < 0.05.
