# Supplementary figures and images for: Silencing of the chemokine CXC receptor 4 (CXCR4) hampers cancer progression and increases cisplatin (DDP)-sensitivity in clear cell renal cell carcinoma (ccRCC)
Source: Bioengineered. 2021 Jun 28;12(1):2957–69. doi: 10.1080/21655979.2021.1943112 (PMC8806489; doi:10.1080/21655979.2021.1943112)

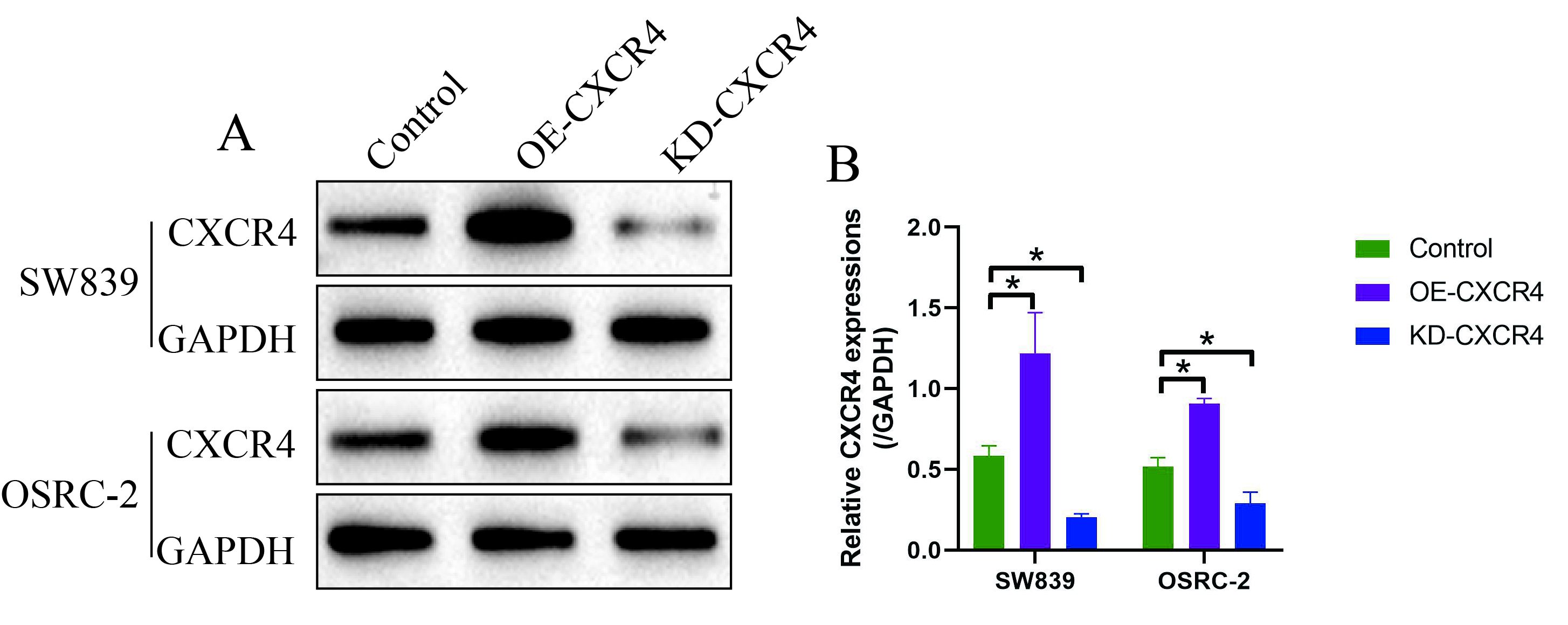

Supplement: Supplemental Material [file KBIE_A_1943112_SM1365.zip › Figure S1.jpg]

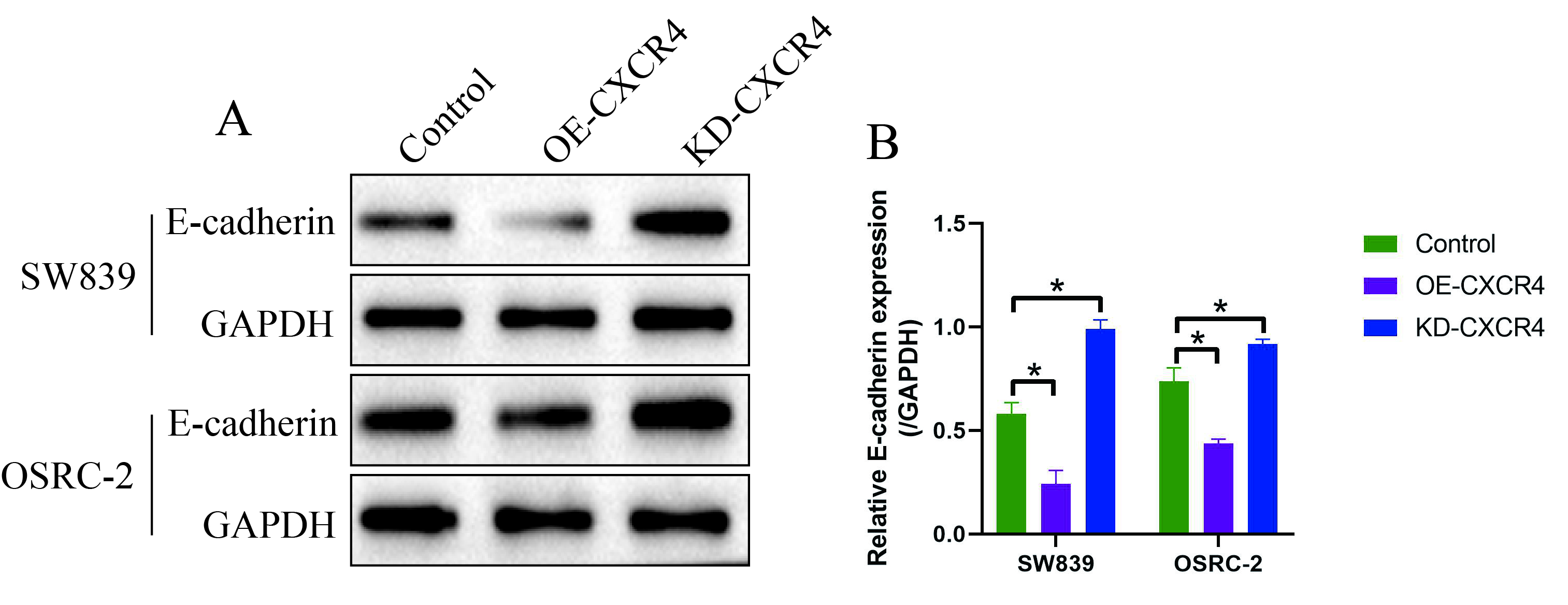

Supplement: Supplemental Material [file KBIE_A_1943112_SM1365.zip › Figure S2.jpg]
